# Application for the Detection of Taste and Smell Disorders Based on Self-Test at the Patient’s Home

**DOI:** 10.3390/diagnostics15243155

**Published:** 2025-12-11

**Authors:** Malgorzata Buksinska, Iwona Tomaszewska-Hert, Malgorzata Talarek, Piotr Henryk Skarzynski

**Affiliations:** 1 Otorhinolaryngosurgery Clinic, World Hearing Center, Institute of Physiology and Pathology of Hearing, 05-830 Kajetany, Poland; m.buksinska@ifps.org.pl; 2 Institute of Sensory Organs, 05-830 Kajetany, Poland; i.tomaszewska@csim.pl (I.T.-H.); p.skarzynski@csim.pl (P.H.S.); 3 Teleaudiology and Screening Department, World Hearing Center, Institute of Physiology and Pathology of Hearing, 05-830 Kajetany, Poland

**Keywords:** taste disorders, smell disorders, self-test

## Abstract

**Background:** Olfactory and taste disorders are associated with a variety of conditions, not only those directly affecting the nasal or oral cavity mucosa. In clinical practice, the acuity of the sense of smell is investigated subjectively using a variety of olfactory tests, both screening and diagnostic. The aim of this study was to develop an application that allows a patient to self-administer an olfactory and taste test at home. **Methods:** In the first stage, a literature review was carried out on the olfactory and taste tests available on the market. In the second stage, the best conditions for storing the ST and TT were investigated. The third stage looked at how well patients of different ages and electronic literacy levels could perform the ST and TT independently. In the fourth stage, the feasibility of using the STT for olfactory and taste screening was assessed. **Results:** The study used an olfactory test based on the ST and TT used in the Sensory Testing Capsule (STC), which can be used for olfactory and taste screening. Development of the app involved steps of registration, ordering, performing the STT, and read-out of results. Simple instructions for performing the STT were created using graphics to illustrate the steps involved. **Conclusions:** The app could also be useful in the assessment of smell and taste by speech therapists before implementing multisensory therapy and used in occupational medicine to monitor the state of the sense of smell and taste in people who are occupationally exposed to toxic substances.

## 1. Introduction

Olfactory and taste disorders (OTDs) are associated with a variety of conditions, not only those directly affecting the nasal or oral cavity mucosa (e.g., rhinosinusitis, chronic sinusitis) but also others such as neurodegenerative disorders, diabetes, thyroid diseases, vitamin deficiencies, medications, surgical interventions of the head and neck, and prior head trauma [1,2].

Olfactory disorders can be divided into quantitative disturbances—such as anosmia (complete loss of smell) and hyposmia (reduced smell sensitivity)—and qualitative disturbances, including parosmia (distorted smell perception) and phantosmia (olfactory hallucinations). Similarly, taste disorders comprise quantitative forms—such as ageusia (complete loss of taste) and hypogeusia (partial loss of taste)—and qualitative disturbances, such as dysgeusia, in which taste perception is distorted or unpleasant [3].

The prevalence of olfactory disorders in the general population is estimated as 12–25%, increasing with age, with approximately 40% of people over 65 years of age experiencing them. Functional anosmia, where a person’s olfactory function is lowered, affects about 5% of the general population [1,2]. The prevalence of taste disorders is 17–19% and also increases with age [4,5]. Both smell and taste dysfunctions can have a substantial impact on quality of life, leading to reduced appetite, weight loss, eating disturbances, and an increased risk of anxiety and depressive symptoms [1,2,6]. In recent years, olfactory and gustatory disorders have gained additional clinical importance due to their association with viral infections such as COVID-19, as well as with neurodegenerative and metabolic diseases, including Parkinson’s and Alzheimer’s disease, diabetes, and thyroid disorders [7,8,9]. Impaired olfactory function may precede neurological symptoms by several years, making smell testing a potential early biomarker in these conditions [10]. Moreover, the loss of smell reduces environmental safety (e.g., impaired detection of smoke or gas leaks) and affects social and emotional well-being, while impaired taste perception can reduce the ability to detect spoiled or contaminated food [11]. These aspects further underscore the need for reliable and accessible screening tools.

In clinical practice, the acuity of the sense of smell is investigated subjectively using a variety of olfactory tests, both screening and diagnostic. Most tests involve the orthonasal olfactory sensation associated with air flowing through the anterior nostrils into the olfactory epithelium. A few tests use the retronasal olfactory sensation caused by flow of air past the soft palate into the nasopharynx, as in swallowing or nasal exhalation. This latter mode is involved when detecting the aroma of food while eating [1,2,12]. Taste function, on the other hand, is typically assessed using solutions or impregnated strips representing the four or five basic tastes (sweet, sour, salty, bitter, and umami). The most common methods include the use of filter paper strips in whole-mouth tests, which measure recognition thresholds or correct identification of taste quality [13]. However, these procedures also require in-person administration by trained examiners, limiting their large-scale use.

Among the most widely used diagnostic tools for evaluating smell and taste function are the University of Pennsylvania Smell Identification Test (UPSIT) and the Burghart ODOFIN™ Sniffin’ Sticks and Taste Strips, which provide standardised and reproducible assessment of both olfactory and gustatory performance [14,15,16]. These methods have become international reference standards in sensory diagnostics and are commonly used in both clinical and research settings.

Recent advances in this field have also led to the development of innovative, automated, and self-administered systems. In Poland, a similar approach has been implemented through the creation of dedicated smell and taste tests integrated into the Sensory Examination Capsule—an automated diagnostic station enabling large-scale screening of smell, taste, hearing, vision, and balance [17]. This solution reflects the ongoing digital transformation of sensory testing and supports the development of remote, app-based diagnostic tools.

To further enhance the accessibility of sensory screening, the smell and taste tests previously implemented in the Sensory Examination Capsule were adapted for use within a mobile application, allowing patients to perform self-administered olfactory and gustatory assessments at home. The present study primarily aims to describe the process of developing this system and to evaluate its applicability for smell and taste screening.

## 2. Materials and Methods

This paper presents the results of a prospective study performed by two consortium collaborators. The study protocol, consent forms, and patient brochure were approved by the Bioethics Committee of the Institute of Physiology and Pathology of Hearing (number BC.IFPS 17/2021) and complied with the World Medical Association’s Declaration of Helsinki. Participation in the study was voluntary and free of charge. All participants provided informed consent prior to inclusion.

Adult patients of the Institute of Physiology and Pathology of Hearing were recruited for the study. Inclusion criteria included signing the informed consent form, being of legal age, and having sufficient physical and mental ability to independently perform the tests and complete the accompanying questionnaires. Individuals who did not provide consent or were unable to complete the questionnaires or perform the smell and/or taste tests independently were excluded.

### 2.1. Measures

*Smell Test (ST)*

The study used an olfactory test based on the smell test (ST) used in the Sensory Testing Capsule (STC) [17], which can be used for olfactory screening. It consists of 6 odour-coated strips encapsulated in microcapsules carrying the scents of cinnamon, banana, smoke, leather, chocolate, and petrol. The strips come in four combinations having different order of the scents. The strips are clipped together in a fan and packaged in an envelope with a QR code, which allows a computer system to recognise the combination and assess the test result. Participants were instructed to scratch each strip, smell it, and select the corresponding answer in the system interface. A result is considered a pass if the patient can identify 5 or 6 odours correctly.

*Sniffin Sticks Test (SST)*

The SST is a common test for assessing the sense of smell. It was originally developed in 1997, and a Polish version was published in 2014 [14,18]. The test uses sticks soaked in different fragrances. During the test, the sticks are brought close to the patient’s nostrils for about 3 s. An extended version of the test consists of three parts, a threshold test, a discrimination test, and an identification test, but in our study, we only used the identification test (iSST). The iSST consists of 16 sticks with different odours. The patient’s task is to indicate the correct scent from among four suggested answers. Each presentation was accompanied by a forced-choice selection panel, and participants were instructed to choose one of the four provided options for every odour. A maximum of 16 points can be obtained in the test. In our study, a score of 12 or more was taken as the norm.

*Taste Test (TT)*

This study used a taste test based on the TT used in the STC [17], which can be used for taste screening. It consists of four paper strips soaked in sweet (40% glucose), salty (25% sodium chloride), bitter (0.6% quinine), and sour (30% citric acid) solutions, as well as one control strip with no taste. The control strip allows the taste and texture of the paper to be familiarised before the actual test begins. The other strips are placed by the patient in the middle of the tongue and removed after about 30 s. The patient’s task is to identify the taste. A correct result is recorded if the patient recognises all samples correctly. All strips are packaged in a cardboard insert, which is placed in an envelope carrying a QR code. The QR code allows the test version number to be identified (4 variants are available) and the test result to be automatically read by a computer.

*Taste Strips (TS)*

TS is a test, validated in 2009, to evaluate the sense of taste based on psychophysical methods [15]. The test kit consists of a set of jars containing strips saturated with substances of four basic tastes (sweet, salty, bitter, sour) in various concentrations. To perform the test, the tester places the strip in the middle of the patient’s tongue and asks them to identify the taste. The manufacturer of the test also offers a version for screening purposes, consisting of strips carrying just the highest concentration of the taste substances, and we used this version in our study. Participants were asked to indicate the perceived taste from four options presented in the answer sheet or system interface. The result was considered correct if the patient correctly identifies all four tastes.

All olfactory and gustatory tests described above were performed in a quiet, well-ventilated room under standardised conditions, following the manufacturer’s recommendations and general guidelines for sensory testing.

### 2.2. Study Plan

**Figure 1:**
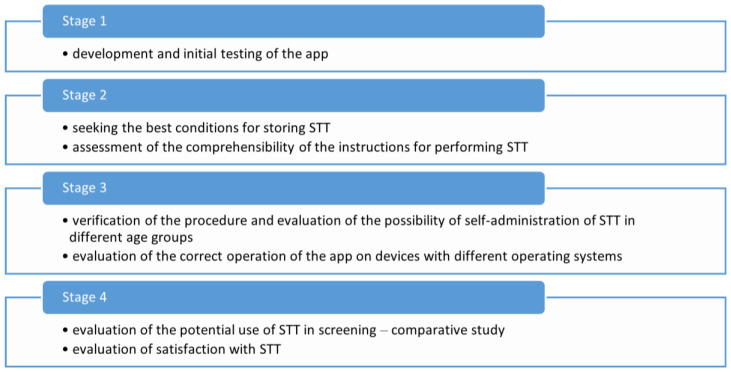
Workflow for creating the app.

The study was divided into four stages, each focusing on a specific aspect of app development, usability testing, and feasibility of self-administered smell and taste screening (Figure 1).

*Stage 1—App concept and prototype testing*

In the first stage, a literature review of the available olfactory and taste tests on the market was carried out. We selected tests that were available in Polish and would allow for self-testing at home, without the need for a researcher or travel to a medical facility [19]. Work then began on developing an app by which the patient could create an individual account, order and perform the ST and/or TT, view the results, and have access to them later. The design phase also included defining the logic of data flow between the mobile interface and the central database, as well as developing algorithms for automatic test result interpretation based on the number of correct identifications. Care was also taken to ensure that registration and data collection complied with current data protection legislation (RODO). To verify the correct functioning of the app, 20 volunteers, who had no olfactory or taste disorders, were asked to perform one ST and one TT. These two tests, when performed together, are hereafter referred to as the STT. The purpose of this stage was to verify the technical functionality of the app prototype and the feasibility of self-administered smell and taste testing.

*Stage 2—Stability of test materials and evaluation of instructions*

The aim of this stage was to determine the optimal storage parameters for the smell and taste tests and to assess the clarity and comprehensibility of the app-based instructions.

Different storage conditions for the STT samples were tested, with each group stored under different conditions: an unventilated room at a temperature of about 28 °C, an air-conditioned room with a temperature of 20 °C, and a refrigerator with a temperature of about 4 °C. After 8 months, a group of 7 healthy people performed an STT from each group and assessed the degree to which the odours and tastes had been retained based on questions about the detectability of the tastes/odours, their intensity, and their identifiability.

A questionnaire was then developed to assess how well the instructions for performing the STT were understood. The questionnaire was given to 30 randomly selected patients of the Institute of Hearing Physiology and Pathology aged 18–65 years who were asked to read the multimedia instructions, register the app, and order the STT. The collected feedback was used to refine the written and multimedia instructions within the application and to ensure user comprehension prior to large-scale deployment.

*Stage 3*

Stage 3 tested how well patients of different ages and levels of electronic literacy could self-administer the STT. Sixty subjects in each of the following age groups were asked to participate: children up to 7 years of age, school children, adolescents, adults up to 50 years of age, adults 50–60 years old, adults 60–70 years old, and adults over 70 (420 subjects in all). The subjects were asked to complete the SST in the presence of a researcher who observed the subject’s behaviour. The researchers assessed whether the instructions and messages in the app were understandable to the user, and whether any of the steps had caused problems. The tests were performed on different devices and different operating systems (MS Windows, Mac OS, Android). The correctness of the QR codes printed on the STT envelopes was also verified.

An additional goal of this stage was to evaluate the usability of the app interface and to determine the success rate, defined as the percentage of users who completed the test without assistance. Observations were recorded using a standardised checklist to ensure consistency of assessment.

*Stage 4*

The fourth stage assessed the feasibility of using STT for olfactory and taste screening. The aim of the work was to gather information on the level of difficulty of performing the STT on a larger group of subjects and to compare results performed using the app with commercially available smell and taste tests (SST and TS). The primary goal of this stage was to evaluate the feasibility, user compliance, and overall performance of the app-based testing in comparison with the reference methods. A total of 1100 people were recruited to take part in the study. In addition, 100 randomly selected patients were asked to complete a questionnaire about their satisfaction with the STT.

## 3. Results

**Stage 1**

**Figure 2:**
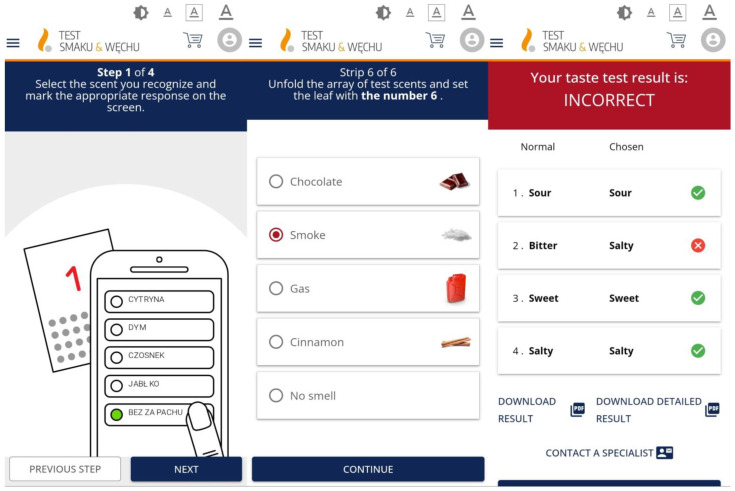
Examples of screenshots from the mobile application. **Left**—instructions for performing the Smell Test (ST); **middle**—selection of the appropriate scent; **right**—example of an incorrect Taste Test (TT) result displayed in the app.

After reviewing the available literature, the feasibility of using different olfactory and taste tests in the app was evaluated, taking into account the time taken to perform a test, how well it performed, and whether it was feasible to mail the test to the patient’s home. A suitable test needed to be performed quickly and able to be performed remotely in the subject’s home without having to travel to a medical facility. Development of the app involved steps of registration, ordering, performing the STT, and read-out of results. Simple instructions for performing the STT were created using graphics to illustrate the steps involved (see Figure 2). The answer form, which appeared on the screen, contained the names of the different smells or tastes, together with icons indicating the correct answer. The result of each test included the names of the selected odour and taste. The results can be saved in pdf format. Figure 2 shows some screenshots.

Initial tests were aimed at assessing the overall performance of the app: its registration, selection of type of test, and whether all steps could be performed satisfactorily. We found that 95% of the olfactory tests and 90% of the taste tests were performed correctly (Table 1).

These preliminary results confirmed that the procedure was intuitive and that users could successfully complete the tests without technical issues. The high completion rate (over 90%) demonstrated that the STT could be effectively performed by non-specialists in a home-like environment, providing a rationale for proceeding to the next stages of system validation.

**Stage 2**

The results of the STT carried out by the group of testers is summarised in Table 2, from which we conclude that suitable conditions for storing STT test kits are an air-conditioned room at a temperature of around 20 °C. Samples stored under these conditions retain their odours and tastes.

On the basis of the group survey, the comprehensibility of the app’s instructions for performing the STT were good, with the instructions clear and easy to use for 29 of 30 people (97%). One person reported difficulties in creating an account due to the complex password required.

These findings confirmed that the stability of both smell and taste samples depends strongly on long-term storage temperature. Maintaining the kits at approximately 20 °C allowed the odours and tastes to remain stable over an 8-month period.

Additionally, feedback from participants indicated that the instructions and on-screen guidance were generally understandable, confirming that the app interface was suitable for independent use.

**Stage 3**

STTs were conducted on 420 people, of whom 404 (96%) performed the test correctly. In terms of age groups, the procedure was performed correctly by at least 90% of the volunteers in each group. Older people, i.e., over 70 years of age, performed STT slightly slower, reflecting less experience with electronic devices. A bug with some operating systems was identified in which the graphical interface did not display as intended; appropriate fixes were made.

These findings demonstrated that the application could be effectively used across different age groups, with only minor usability challenges observed among the oldest participants. The high completion rate confirmed the suitability of the app-based procedure for independent use in diverse populations.

**Stage 4**

Some 1100 complete test results were obtained (SST, TS, ST, and TT), based on which the potential of the ST and TT performed with the Application for use in clinical practice as screening tests could be assessed. Table 3 indicates that satisfactory parameters were obtained. Both tests demonstrated high diagnostic accuracy, with true positive and true negative rates exceeding 90%, confirming a good level of agreement with the reference methods (SST and TS).

The results of the Smell Test (ST) and Taste Test (TT) were analysed by gender. Table 4 shows the distribution of test results by gender. Women achieved higher scores than men in both tests—61.0% vs. 56.3% for smell and 69.7% vs. 60.2% for taste. The difference was more pronounced for the Taste Test (TT), indicating slightly better gustatory performance among female participants.

The results were also analysed by age group. Table 5 shows the distribution of test results across age groups. The highest proportion of correct results in both tests was observed in participants aged 19–35 years (75.1% for ST and 73.5% for TT), followed by the 36–59 group. The lowest scores were obtained in the oldest age group (≥60 years), where only 38.3% correctly identified smells and 52.2% correctly identified tastes. Both tests revealed an age-related decline in performance, which was more pronounced for olfactory identification.

**Figure 3:**
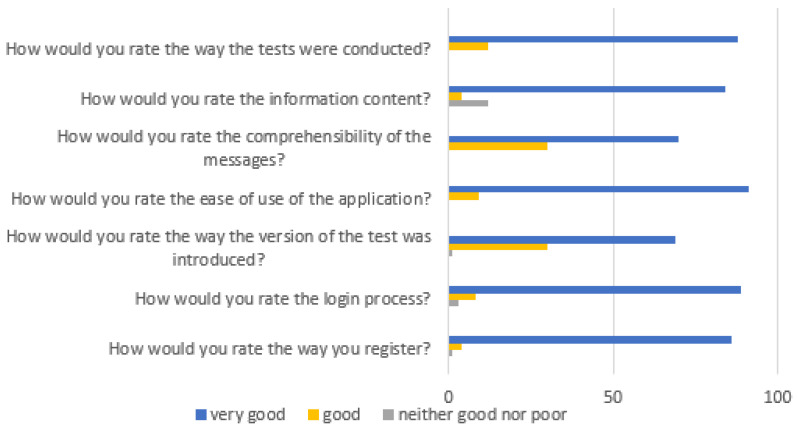
Results of the satisfaction survey.

Based on the responses to the satisfaction survey, we conclude that the app was easy to use for most respondents, the messages in it were understandable, and the information displayed was of interest. In addition, the majority of users described the interface as clear and intuitive, and no major technical difficulties were reported during testing. The results of the survey are shown in Figure 3.

Overall, the obtained results confirmed the feasibility of using the developed application for olfactory and gustatory self-assessment. Both the performance data and user feedback support its potential use as a practical screening tool for large-scale sensory testing. These findings formed the basis for further analysis and interpretation discussed in the following section.

## 4. Discussion

Telemedicine makes it possible to perform various tests without the patient having to travel to a medical facility. So far, a screening tool for the sense of smell that can be performed independently at home by the patient without the need for contact with medical staff has not been described. In this study, we chose the previously used ST and TT as tests that could be used in conjunction with the newly developed mobile application. Our findings demonstrated that the proposed digital approach allows reliable and reproducible assessment of smell and taste in a home setting, showing high diagnostic accuracy compared with standard reference methods.

Traditionally, the ST and TT require travel to a medical centre where the sense of smell and taste can be tested, either alone or with the help of staff. In many cases, the need for assistance may arise due to medical conditions (chronic conditions or acute infections requiring isolation) or socioeconomic conditions (distance from home, travel time and cost). Building STT into an app allows olfactory and/or taste tests to be performed in the patient’s home. For children or people with physical or intellectual disabilities, the test could be performed with the help of a carer.

During the development process, particular attention was given to the clarity of the instructions, ease of use, and reliability of the application. The conducted usability studies demonstrated that the application is intuitive and user-friendly. Minor difficulties were observed mainly among elderly participants, who generally have less experience with modern technologies; however, they constituted a small percentage of users. Similar findings have been reported previously, indicating that older adults tend to perform less efficiently in digital health assessments due to reduced technology experience rather than sensory limitations [20,21].

In parallel, storage stability experiments were performed to determine the optimal environmental conditions for maintaining the integrity of the smell and taste samples. The results confirmed that both olfactory and gustatory strips remain stable and retain their sensory properties when stored at approximately 20 °C, whereas exposure to higher or lower temperatures led to partial degradation. These conditions were therefore included in the information leaflet accompanying each test set to ensure reproducibility and test quality. The findings confirm that the test materials can be safely distributed to patients by mail, which is critical for large-scale home-based screening.

The effectiveness of the application in detecting olfactory and gustatory disorders was also evaluated by comparing the results of the ST and TT with reference tests SST and TS. Analysis of over 1100 complete test results showed a high level of agreement between the app-based screening tests and the standard diagnostic procedures. The obtained diagnostic parameters (TPR, TNR, PPV, NPV) confirmed that both the ST and TT exhibit high sensitivity and specificity, indicating their usefulness in clinical practice. These findings demonstrate that mobile technology can provide a reliable alternative to conventional screening methods for assessing the sense of smell and taste. Identification-based smell and taste tests inherently engage semantic memory, which makes them particularly effective for detecting central chemosensory deficits such as those associated with neurodegenerative diseases. However, this characteristic also limits their sensitivity in cases of mild peripheral hyposmia, for example, in patients experiencing upper respiratory tract infections, allergic inflammation or exposure to toxic substances. These factors should be considered when interpreting screening results obtained with the ST and TT, especially in populations at risk of transient or environmentally induced olfactory loss.

In addition to overall diagnostic accuracy, we also analysed the distribution of results by gender and age (Table 4 and Table 5). The data showed that women achieved higher accuracy rates than men in both smell and taste tests, which is consistent with previous reports indicating better olfactory and gustatory performance among females [22,23,24].These differences have been attributed to hormonal influences, greater sensory attention, and potentially higher receptor sensitivity.

When considering age, our results confirmed a progressive decline in smell and taste performance with increasing age. Similar trends have been described in population studies, where sensory decline has been linked to cumulative effects of upper respiratory infections, chronic rhinosinusitis, head trauma, environmental exposure, and neurodegenerative changes [24,25,26]. These differences have been attributed to hormonal influences, greater sensory attention, and odor sensitivity [26,27]. In contrast, the lower recognition rates observed in the youngest group may reflect limited sensory experience and incomplete development of odour and taste identification templates [28,29]. These findings indicate that paediatric assessment requires age-appropriate reference values and stimuli adapted to children’s daily sensory exposure. Younger children may also show reduced familiarity with certain odorants that are commonly used in adult smell tests, which may further contribute to lower identification scores independently of true olfactory function. Validated paediatric smell tests typically use odorants selected specifically for children [30], and this aspect should be considered when interpreting results in the youngest age group.

Furthermore, user satisfaction was assessed to evaluate the overall usability of the application. The results of the satisfaction survey (Figure 3) showed that most participants rated the app as easy to use, clear, and visually accessible. These findings confirm that the interface and instructional design were appropriate for independent use. Similar observations have been reported in previous studies on user-centred mobile health applications, indicating that intuitive and well-structured interfaces improve engagement and adherence [31].

By using the application and comparing results over time, it is possible to monitor the sense of smell and taste over the course of nasal and sinus disease, upper respiratory tract infection, and neurodegenerative conditions. The app could also be useful in the assessment of smell and taste by speech therapists before implementing multisensory therapy and used in occupational medicine to monitor the state of the sense of smell and taste in people who are occupationally exposed to toxic substances.

Overall, the obtained findings indicate that digital self-assessment tools such as the developed application can be successfully incorporated into modern telemedicine systems. High completion rates and positive user feedback confirm their feasibility for large-scale implementation. Future research should explore longitudinal monitoring and integration with electronic medical records to improve early detection and preventive strategies for sensory disorders.

## 5. Limitations

This study has several limitations. First, the tests included in the application rely exclusively on odour and taste identification tasks and therefore do not assess olfactory or gustatory thresholds. As a result, the tool may be less sensitive in detecting mild peripheral hyposmia, for example, that is related to infection, allergic inflammation or mucosal oedema, conditions that typically affect detection thresholds before identification ability. In addition, identification tasks engage semantic memory, making them more sensitive to central chemosensory deficits (e.g., neurodegenerative disorders) than to subtle peripheral loss.

Second, the same odorants were used for adults and children. Reduced familiarity with certain smells may have contributed to lower identification scores among younger participants, independently of true olfactory function. Validated paediatric smell tests typically use odorants specifically selected for children, reflecting their everyday sensory exposure and developmental experience. Future versions of the test should therefore include age-appropriate odour sets to improve ecological validity in paediatric screening.

## 6. Conclusions

The developed mobile application enables convenient, self-administered screening of smell and taste function in a home environment using short identification tasks. The results of this study demonstrate high diagnostic agreement between the app-based screening tests (ST and TT) and established reference methods (SST and TS), confirming that the proposed digital tool provides a reliable alternative for rapid chemosensory assessment. Its usability and acceptability across different age groups further support its potential for large-scale implementation.

The application may be used for population-based screening; longitudinal monitoring of patients with nasal and sinus disease, upper respiratory tract infections or neurodegenerative conditions; and for evaluating chemosensory function in occupational settings. The tool also offers new opportunities for remote assessment within telemedicine pathways, supporting early detection and follow-up without requiring in-person clinical visits.

Future development should include incorporating age-appropriate odorants for children and expanding test procedures beyond identification tasks (e.g., threshold or discrimination assessments) to improve sensitivity for mild peripheral olfactory loss. Integrating the application with digital medical records and automated alert systems may further enhance its value in preventive and personalised medicine.

## Figures and Tables

**Table 1:** Results obtained from the app using the Taste and Smell Tests.

Type of Test	Number of People Tested	Number of Tests Performed Correctly	% of Tests Performed Correctly
Smell Test (ST)	20	18	90
Taste Test (TT)	20	19	95

**Table 2:** Results of STT after different storage conditions.

Type of Test	Storage of Samples	% Correct Answers	Comments
Taste Test (TT)	Unventilated room with temperature of approximately 28 °C	75	Tests stored above about 28 °C lose their properties despite being packed in an airtight ziplock bag. Elevated temperature causes the flavouring substances to degrade
	Room with air conditioning at 20 °C	100	100% of test samples tested did not change their properties or lose quality
	Very cool room—fridge temp 4 °C	80	Low storage temperatures adversely affect the quality of the tests, weakening the intensity of the flavouring substances
Smell Test (ST)	Unventilated room with a temperature of approximately 28 °C	80	Tests stored at approx. 28 °C lose their properties despite being packaged in an airtight ziplock bag. High temperatures cause the microcapsules with the fragrance to burst, making the fragrances unrecognisable
	Room with air conditioning at 20 °C	100	100% of test samples did not change their properties or lose quality
	Very cool room—fridge temp 4 °C	85	Too low a storage temperature adversely affects the quality of the tests. A cold temperature of around 4 degrees causes the microcapsules with the fragrance to shrink and break, rendering the test unusable.

**Table 3:** Parameters reflecting the accuracy of the SST (*n* = 1100).

	ST	TT
True Positive (TP)	381	311
False Positive (FP)	67	61
True Negative (TN)	611	709
False Negative (FN)	41	19
True Positive Rate (TPR)	90.2%	94.2%
True Negative Rate (TNR)	90.1%	92.1%
Positive Predictive Value (PPV)	85.0%	83.6%
Negative Predictive Value (NPV)	93.7%	97.4%

**Table 4:** Results of the Smell Test (ST) and Taste Test (TT) by gender (*n* = 1100).

Gender	Number of Participants (*n*)	ST—Correct Results (%)	ST—Incorrect Results (%)	TT—Correct Results (%)	TT—Incorrect Results (%)
Women	693	61.0	39.0	69.7	30.3
Men	407	56.3	43.7	60.2	39.8
Total	1100	59.3	40.7	66.2	33.8

**Table 5:** Results of the Smell Test (ST) and Taste Test (TT) by age group (*n* = 1100).

Age Group (Years)	ST—Correct Results (%)	ST—Incorrect Results (%)	TT—Correct Results (%)	TT—Incorrect Results (%)
<18	52.7	47.4	65.3	34.7
19–35	75.1	24.9	73.5	26.5
36–59	66.8	33.3	71.5	28.5
≥60	38.3	61.7	52.2	47.8
Total	59.3	40.7	66.2	33.8

## Data Availability

The raw data supporting the conclusions of this article will be made available by the authors on request.

## Abbreviations

The following abbreviations are used in this manuscript:

OTDs	Olfactory and taste disorders
STC	Sensory Testing Capsule
ST	Smell Test
STT	Sniffin Sticks Test
TT	Taste Test
TS	Taste Strip
STT	Smell Taste Test
